# Arg354 in the catalytic centre of bovine liver catalase is protected from methylglyoxal-mediated glycation

**DOI:** 10.1186/s13104-015-1793-5

**Published:** 2015-12-30

**Authors:** Christian Q. Scheckhuber

**Affiliations:** Senckenberg Research Institute, LOEWE Excellence Cluster for Integrative Fungal Research (IPF), Georg-Voigt-Str. 14-16, 60325 Frankfurt, Germany

**Keywords:** Arginine, Bovine liver catalase, Dihydroxyimidazolidine, Glycation, Hydroimidazolone, Methylglyoxal, Modification

## Abstract

**Background:**

In addition to controlled post-translational modifications proteins can be modified with highly reactive compounds. Usually this leads to a compromised functionality of the protein. Methylglyoxal is one of the most common agents that attack arginine residues. Methylglyoxal is also regarded as a pro-oxidant that affects cellular redox homeostasis by contributing to the formation of reactive oxygen species. Antioxidant enzymes like catalase are required to protect the cell from oxidative damage. These enzymes are also targets for methylglyoxal-mediated modification which could severely affect their catalytic activity in breaking down reactive oxygen species to less reactive or inert compounds.

**Results:**

Here, bovine liver catalase was incubated with high levels of methylglyoxal to induce its glycation. This treatment did not lead to a pronounced reduction of enzymatic activity. Subsequently methylglyoxal-mediated arginine modifications (hydroimidazolone and dihydroxyimidazolidine) were quantitatively analysed by sensitive nano high performance liquid chromatography/electron spray ionisation/tandem mass spectrometry. Whereas several arginine residues displayed low to moderate levels of glycation (e.g., Arg93, Arg365, Arg444) Arg354 in the active centre of catalase was never found to be modified.

**Conclusions:**

Bovine liver catalase is able to tolerate very high levels of the modifying α-oxoaldehyde methylglyoxal so that its essential enzymatic function is not impaired.

**Electronic supplementary material:**

The online version of this article (doi:10.1186/s13104-015-1793-5) contains supplementary material, which is available to authorized users.

## Background

Proteins can be covalently modified by a staggering variety of chemical moieties. Some of these modifications lead to changes in the functional life time of a protein (e.g., polyubiquitination as a prerequisite for degradation of the target protein by the 26 s proteasome, SUMOylation for enhancing protein stability, etc.). Other modifications more or less directly influence enzymatic activity (e.g., phosphorylation and acetylation to name but a few). The aforementioned examples have in common that the cell controls if or if not a given post-translational modification occurs. In contrast, modifications that occur non-enzymatically are often detrimental to protein function (although there are exceptions from this rule) [1]. Among these are reactions of highly reactive low molecular weight compounds (e.g., reactive oxygen species, reducing sugars, etc.) with specific amino acids [2, 3]. Here the reaction of methylglyoxal with arginine (and to a lesser extent lysine) residues will be briefly described. Methylglyoxal is formed as a toxic by-product of the catabolic breakdown of d-glucose (glycolysis) [4]. Usually the cellular level of this α-oxoaldehyde is rather low (approx. 2–4 µM in cells and tissues [5–7]). However, in pathological hyperglycaemic conditions like diabetes it can be elevated to critical levels and subsequently severely impair cellular functions [8–10]. After the initial binding of methylglyoxal to the protein hydroimidazolone MG-H1 and dihydroxyimidazolidine (DHI) [11] intermediates are formed that can be further rearranged to give rise to so-called ‘advanced glycation end products’ (AGEs) [12]. The deleterious properties of AGEs are well documented and many efforts are directed to counteract their formation (reviewed in [13]). Methylglyoxal-mediated toxicity is intimately linked to conditions of oxidative stress (reviewed in [14]). These two damaging situations amplify each other and unsurprisingly cause profound obstacles for cellular function and survival. Methylglyoxal is known to affect the ability of a cell to cope with oxidative stress by modifying antioxidative proteins like catalase [15, 16], ascorbate peroxidase [17], superoxide dismutase [16], glutathione peroxidase [18] and albumin [19] which are required to keep levels of reactive oxygen species at (low) physiological concentrations. Catalase isolated from bovine liver is a protein of 240 kDa (mass of the functional tetramer). Heme serves as a prosthetic group while NADPH can be bound as a cofactor. The former is required for the catalytic breakdown of the reactive oxygen species hydrogen peroxide to water and molecular oxygen [20] whereas the latter seems to protect the enzyme from off-pathway oxidized enzyme states [21, 22]. An amino acid residue of critical importance is Arginine354 (Arg354) which constitutes a ‘charge relay’ along with His218 and Asp348 to stabilize the electrostatic field generated by different iron oxidation states within the active centre of the enzyme [22]. It has been speculated that the modification of this arginine by methylglyoxal-mediated glycation could lead to an altered catalase activity [15].

To test whether Arg354 is a target for glycation by methylglyoxal experimentally an in vitro glycation procedure was developed. Unlike previous protocols this procedure uses relatively high concentrations of methylglyoxal (160 mM) for short periods of time (2 h). In order to identify whether Arg354 is glycated or not nanoHPLC-ESI–MS/MS was performed. In addition to Arg354 other arginine residues involved in the formation of catalase tetramers and in NADPH binding were investigated. The data presented here suggest that Arg354 is protected from methylglyoxal-mediated glycation. This is in line with measurements of catalase activity demonstrating that methylglyoxal treatment has no significant effect on the catalytic activity of catalase. However, other arginines (Arg93, Arg263, Arg365 and Arg444) display low to medium levels of MG-H1 and DHI formation indicating that glycation occurred at these positions. In summary, bovine liver catalase is able to maintain its activity under experimental conditions involving high levels of methylglyoxal that lead to glycation of several arginine residues but not of Arg354 which is located in the active centre of the enzyme. These findings demonstrate the importance of this antioxidative enzyme in keeping levels of hydrogen peroxide low even under challenging conditions.

## Results

### Effects of methylglyoxal treatment on bovine liver catalase activity

The effect of methylglyoxal on the enzymatic activity of bovine liver catalase has been investigated in a previous study [15]. In the present work, an altered protocol for in vitro glycation of this enzyme [higher methylglyoxal concentrations (160 mM vs. up to 5 mM) for shorter times (2 h) instead of at least 5 h] has been applied. The aim of these experiments was not to emulate in vivo levels of methylglyoxal but to determine whether different arginine residues of bovine liver catalase display quantitative differences in their tendency to be subject to glycation. In order to determine catalase activity under these experimental glycation conditions a spectrophotometric assay (see “Methods” section) was used.

**Fig. 1:**
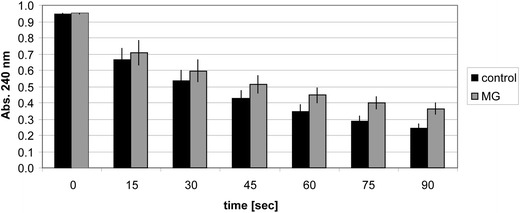
Determination of catalase activity in the absence or presence of methylglyoxal. Mean values of three different measurements are shown in the diagram. *Error bars* denote the standard deviation of the mean value. *MG* methylglyoxal (160 mM)

A catalase-containing solution that has not been treated with methylglyoxal is capable to degrade half of the hydrogen peroxide present in the assay buffer at time 0 within approx. 45 s (Fig. 1). When the purified catalase enzyme is pre-incubated in buffer containing 160 mM methylglyoxal for 2 h before being diluted into assay buffer no clear differences of enzyme activity compared to the untreated control are measured. Catalase activity is maintained at later time points of the assay (60, 75, 90 s) although it tends to be slightly reduced compared to the untreated control. These data show that bovine liver catalase is able to maintain its activity even in the presence of high concentrations of methylglyoxal.

### Analysis of methylglyoxal-mediated protein modifications

The question arises if and to what extent relevant arginine residues in bovine liver catalase have been subjected to glycation during the in vitro glycation experiment. A highly sensitive method (nanoHPLC-ESI–MS/MS, see “Methods” section) has been utilized to analyse catalase peptide ions for modifications of arginine residues. The total ion currents and extracted ion currents of the mass spectra corresponding to the modifications MG-H1 and DHI have been compiled in Additional file 1: Figure S1. The following functionally relevant arginine residues [22] were quantitatively analysed for modifications: Arg66, Arg68, Arg263, Arg365 are involved in the formation of the catalase tetramer interface, Arg354 is involved in binding of the heme prosthetic group in the active centre of the enzyme, Arg203 contributes to binding of the cofactor NADPH while Arg93 and Arg444 have no documented role in catalase function and serve as “out-groupers”.

Both Arg93 and Arg444 display the highest levels of glycation modifications (Table 1) (Arg93: 13.2 % of peptide ions bear the MG-H1 modification and 10.8 % are DHI-modified; Arg444: 6.3 % MG-H1 and 7.3 % DHI). These arginine residues seem to be highly accessible to the reaction with methylglyoxal (see “Discussion” section). Furthermore, this result shows that MG-H1 and DHI modifications occur using the glycation protocol used in this study (“glycation positive control”). It was not possible to obtain peptide ions which solely contain Arg203 and no other arginine (Table 1). The peptide ion that was analysed contains a further arginine residue (Arg210). Therefore the detected modifications (MG-H1: 2.7 %, DHI: 1.0 %) cannot be solely attributed to Arg203. However, it is clear that the level of modification is much smaller compared to the out-groupers. The three arginine residues involved in the formation of the tetramer interaction surface all display low levels of modification (Arg66 + Arg68, MG-H1: 1.4 %; DHI: 1.9 %; Arg263, MG-H1: 0.1 %; DHI: 0.4 %; Arg365, MG-H1: 1.6 %; DHI: 0.9 %) (Table 1). Strikingly, Arg354 located in the active centre of catalase displays no modifications (neither MG-H1 nor DHI) at all (Table 1). In summary these data indicate that bovine liver catalase is able to maintain integrity of its catalytic core even in the presence of very high levels of methylglyoxal.

**Table 1:** Methylglyoxal-dependent modifications of catalase

Sequence	Position	Modification	m/z	Peak area	% modified
F.D*R*E*R*IPERVVH.A	65–75	nm	469.260	141,239,376	
		MG-H1	487.263	1,972,739	1.4
		DHI	493.267	2,816,325	1.9
F.GYFEVTHDIT*R*Y.S	83–94	nm	750.860	35,923,631	
		MG-H1	777.865	6,243,848	13.2
		DHI	786.870	5,107,276	10.8
F.SD*R*GIPDGH*R*HM.N	201–212	nm	459.886	36,013,455	
		MG-H1	477.889	1,015,794	2.7
		DHI	483.893	1,049,274	1.0
L.AHEDPDYGL*R*DLF.N	254–266	nm	774.360	104,144,627	
		MG-H1	801.365	140,068	0.1
		DHI	810.370	393,409	0.4
M.LQG*R*LF.A	351–356	nm	367.221	68,678,448	
		MG-H1	394.226	nd	0.0
		DHI	403.231	nd	0.0
H.*R*H*R*LGPNYLQIPVNCPY.R	363–379	nm	699.698	13,777,078	
		MG-H1	717.702	225,543	1.6
		DHI	723.705	130,193	0.9
F.NSANDDNVTQV*R*TF.Y	433–446	nm	790.868	194,414,574	
		MG-H1	817.874	14,193,861	6.3
		DHI	826.879	16,307,423	7.3

Arginine residues of interest are printed in italic letters

*m/z* mass to charge ratio, *nm* non-modified catalase peptide ion, *nd* not detected

## Discussion

Although harsh glycation conditions were used for the treatment of bovine liver catalase arginine residue 354 in the catalytic centre of this enzyme was not found to be modified by methylglyoxal. Bovine liver catalase activity can indeed be maintained under these conditions which is in line with a previous study that used much lower amounts of glycating agents [15]. Arg354 is situated in a deep pocket formed by a β-barrel structure with few channels providing access to the heme. This arginine residue is involved in the catalytic mechanism of breaking down the catalase substrate, hydrogen peroxide, by cleavage of the peroxide bond. This leads to the formation of an intermediate compound with subsequent neutralisation of Arg354 [22]. The data presented in this study suggest that the reactive methylglyoxal is not able to penetrate this deep into the active centre of the enzyme. By contrast, Arg93 and Arg444 show the highest level of methylglyoxal-mediated modification. This finding correlates well with the fact that these residues are located on the surface of the protein according to the crystal structure of bovine liver catalase as determined by electron crystallography of thin 3D crystals (Protein Data Bank Europe identifier 3j7u) [23] and thus accessible to methylglyoxal. Arg66, Arg68, Arg203, Arg210, Arg263 and Arg365 are neither buried deep in hydrophobic pockets nor directly located on the surface of catalase and display low to moderate levels of glycation.

Certainly it is important that key antioxidative enzymes like catalase are protected from deleterious glycation reactions because it is well documented that methylglyoxal can contribute to oxidative stress in various cell types (reviewed in [14]). For instance, methylglyoxal was found to elevate the generation of hydrogen peroxide and the pro-oxidant peroxynitrite in rat aortic vascular smooth muscle cells [24]. Not only hydrogen peroxide levels were increased by methylglyoxal in these cells but also other reactive oxygen species like superoxide anions were present in elevated concentrations. Methylglyoxal was also found to deplete cellular stores of the antioxidant molecule glutathione (GSH) which is of high importance for maintenance of the cellular redox potential [25, 26]. There are many more examples how methylglyoxal negatively influences the cellular redox balance (reviewed in [14]). In summary, methylglyoxal can be classified as a pro-oxidant. Without the defensive function of antioxidative enzymes like catalase organisms would be rendered even more susceptible to oxidative stress and its deleterious consequences for health and viability. Therefore it seems plausible that the catalytic centre of catalase (specifically Arg354) is protected from methylglyoxal-mediated glycation. The activity of other antioxidant enzymes is also quite resistant to modification by glycating agents (e.g., incubation of superoxide dismutase with 50 mM fructose for 3 days led to >80 % remaining enzymatic activity [16]). By contrast, ascorbate peroxidase from *Nicotiana tabacum* displays activity loss when this protein is subject to glycation [17]. This is probably due to an increased km value for ascorbate due to the glycation [17]. In another study, glutathione peroxidase activity was found to be severely affected by treatment with 5 mM methylglyoxal for 3 h [18]. These results suggest that peroxidases are more readily affected by methylglyoxal compared to catalase and superoxide dismutase.

## Methods

### Catalase glycation procedure/catalase activity assay

Catalase (cat. no. C1345, catalase from bovine liver, Sigma–Aldrich, Taufkirchen, Germany) stock solution (10 mg/ml) was prepared in 50 mM potassium phosphate buffer (pH 7.0) and stored at 4 °C. Before beginning the assays, the stock solution was diluted 1/100 in 50 mM potassium phosphate buffer (pH 7.0) containing no (control) or 160 mM methylglyoxal (cat. no. M0252, Sigma-Aldrich, Taufkirchen, Germany) for 2 h at room temperature. 100 µl of this solution was mixed with 2.9 ml assay buffer (0.075 % [v/v] hydrogen peroxide in 50 mM potassium phosphate buffer [pH 7.0]). Measurements were made exactly for 90 s after enzyme addition in 15 s intervals at 240 nm in a spectrophotometer (NanoPhotometer, Implen, Munich, Germany). As a negative control, 0.075 % (v/v) hydrogen peroxide in 50 mM potassium phosphate buffer (pH 7.0) was incubated with 160 mM methylglyoxal without added catalase. There was no change in absorbance at 240 nm (i.e., it remained constant at approx. A_240nm_ 0.93). This control demonstrates that hydrogen peroxide and methylglyoxal do not react non-enzymatically with each other.

### Determination of methylglyoxal-mediated arginine modifications of catalase

Catalase samples treated with methylglyoxal as described above and untreated controls were purified by using Amicon Ultra-0.5 30 K centrifugal filter devices (Merck Millipore, Schwalbach, Germany). The washing step was conducted with pure water. The purified catalase solution (ca. 50 µl) was added to 50 µl 100 mM potassium phosphate buffer, pH 6.9.

Characterisation of arginine modifications in control and methylglyoxal-treated catalase via nanoHPLC-ESI–MS/MS was performed by Proteome Factory AG (Berlin, Germany). Dry protein samples were dissolved in 8 M urea containing 25 mM TCEP [Tris(2-carboxyethyl)phosphine] and incubated for 30 min at room temperature. Alkylation was carried out for an additional 30 min by adding 50 mM iodoacetamide (final concentration). The alkylated protein samples were subject to proteolysis over-night using chymotrypsin 1:50 (w/w) (Sigma, Germany). The acidified peptides were analysed by nanoHPLC-ESI–MS/MS. The liquid chromatography mass spectrometry system consisted of an Agilent 1100 nanoHPLC (Agilent, Waldbronn, Germany), PicoTip electrospray emitter (New Objective, Woburn, MA) and an Orbitrap XL or LTQ-FT Ultra mass spectrometer (ThermoFisher Scientific, Bremen, Germany). Peptides were first trapped and desalted on the enrichment column (Zorbax 300SB-C18, 0.3 × 5 mm, Agilent) for 5 min (solvent: 2.5 % acetonitrile/0.5 % formic acid), then separated on a Zorbax 300SB-C18, 75 µm × 150 mm column (Agilent) using a linear gradient from 10 to 32 % B (solvent A: 5 % acetonitrile in water, solvent B: acetonitrile, both with 0.1 % formic acid). Ions of interest were subjected to MS/MS according to the expected charge state distribution of peptide ions. Proteins were identified by database search against the NCBInr protein database (National Center for Biotechnology Information, Bethesda, USA) using MS/MS ion search of the Mascot search engine (Matrix Science, London, England) allowing detection of the glycation modifications at arginine residues dihydroxyimidazolidine (DHI) (peptide mass shift: +72 Da) and hydroimidazolone MG-H1 (peptide mass shift: +54 Da), respectively. Selected ion signals were extracted using Qualbrowser (ThermoFisher Scientific, Bremen, Germany).

### Calculation of methylglyoxal-modified peptide ions

The peptide ions that were detected with the highest abundance were selected for calculating the percentage of methylglyoxal-mediated MG-H1 and DHI modifications. The areas of peaks of interest in the chromatograms were determined by integration. Calculations for % modifications of peptide ions were performed with the following equations:

1 $$ {\text{MGH1}} \,[\% {\text{mod}}] = \left( {\frac{{{\text{A}_{\text{MGH1}}}}}{{{\text{A}_{\text{MGH1}}} + {\text{A}_{\text{DHI}}} + {\text{A}_{\text{NM}}}}}} \right) \times 100\,\%  $$

2 $$ {\text{DHI}}\,[\% {\text{mod}}] = \left( {\frac{{{\text{A}_{\text{DHI}}}}}{{{\text{A}_{\text{MGH1}}} + {\text{A}_{\text{DHI}}} + {\text{A}_{\text{NM}}}}}} \right) \times 100\,\%  $$

A_DHI_ and A_MGH1_ correspond to the areas of the integrated peaks of the DHI and MG-1 modified peptide ions, respectively. A_NM_ is the area of the integrated peak of the non-modified peptide ion.

## Additional file

10.1186/s13104-015-1793-5 Raw data for the quantitative determination of arginine residue modifications of bovine liver catalase treated without or with 160 mM methylglyoxal (total ion currents and extracted ion currents of the mass spectra corresponding to non-modified and modified peptide ions [MG-H1 and DHI]).

## Abbreviations

AGE: advanced glycation end product
DHI: dihydroxyimidazolidine
MG-H1: hydroimidazolone
