# Supplementary material for: Arg354 in the catalytic centre of bovine liver catalase is protected from methylglyoxal-mediated glycation
Source: BMC Res Notes. 2015 Dec 30;8:830. doi: 10.1186/s13104-015-1793-5 (PMC4696219; doi:10.1186/s13104-015-1793-5)
Supplement: Supplementary file 1 — 10.1186/s13104-015-1793-5 Raw data for the quantitative determination of arginine residue modifications of bovine liver catalase treated without or with 160 mM methylglyoxal (total ion currents and extracted ion currents of the mass spectra corresponding to non-modified and modified peptide ions [MG-H1 and DHI]). [file 13104_2015_1793_MOESM1_ESM.pdf]

Supplementary information

Arg354 in the catalytic centre of bovine liver catalase is protected from methylglyoxal-mediated glycation

Christian Q. Scheckhuber*

** Corresponding author contact details:*
Senckenberg Research Institute
LOEWE Excellence Cluster for Integrative Fungal Research (IPF)
Georg-Voigt-Str. 14-16
D-60325 Frankfurt am Main
Germany

Email: c.scheckhuber@gmail.com

Fig. S1 (next pages). Raw data for the quantitative determination of arginine residue modifications of bovine liver catalase treated without or with 160 mM methylglyoxal (total ion currents and extracted ion currents of the mass spectra corresponding to non-modified and modified peptide ions [MG-H1 and DHI]).

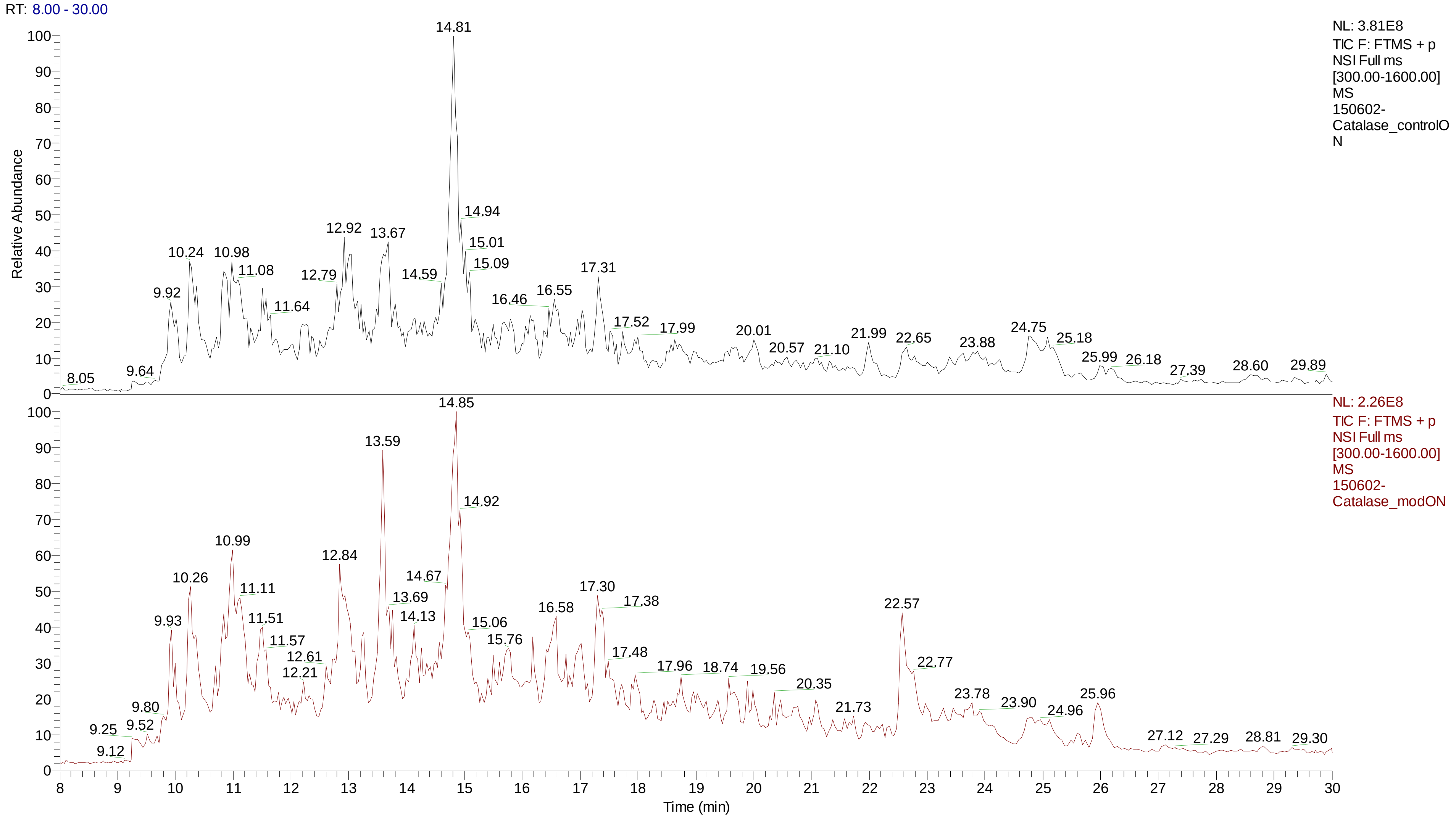

TICs of catalase (control) vs catalase (modified)

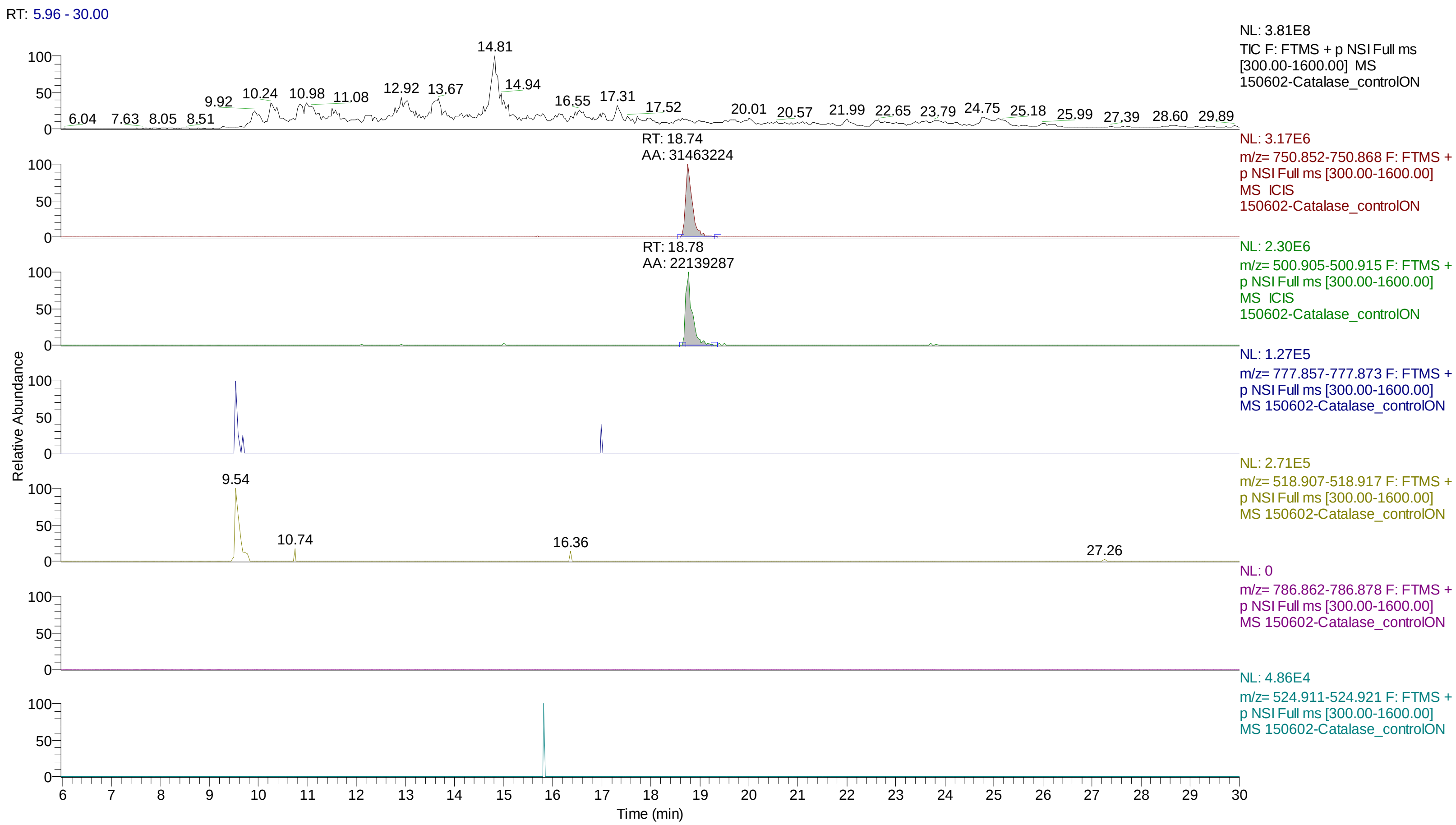

Fig. AA 83-94 in sample catalase (control), top to bottom:

83 - 94 750.8597 F.GYFEVTHDITRY.S
83 - 94 500.9095 F.GYFEVTHDITRY.S
83 - 94 777.8653 F.GYFEVTHDITRY.S MG-H1 (R) false positive signal at RT=9.5 min
83 - 94 518.9116 F.GYFEVTHDITRY.S MG-H1 (R) false positive signal at RT=9.5 min
83 - 94 786.8702 F.GYFEVTHDITRY.S Dihydroxyimidazolidine (R)
83 - 94 524.9158 F.GYFEVTHDITRY.S Dihydroxyimidazolidine (R)
Fig. AA 83-94 in sample catalase (control), top to bottom:

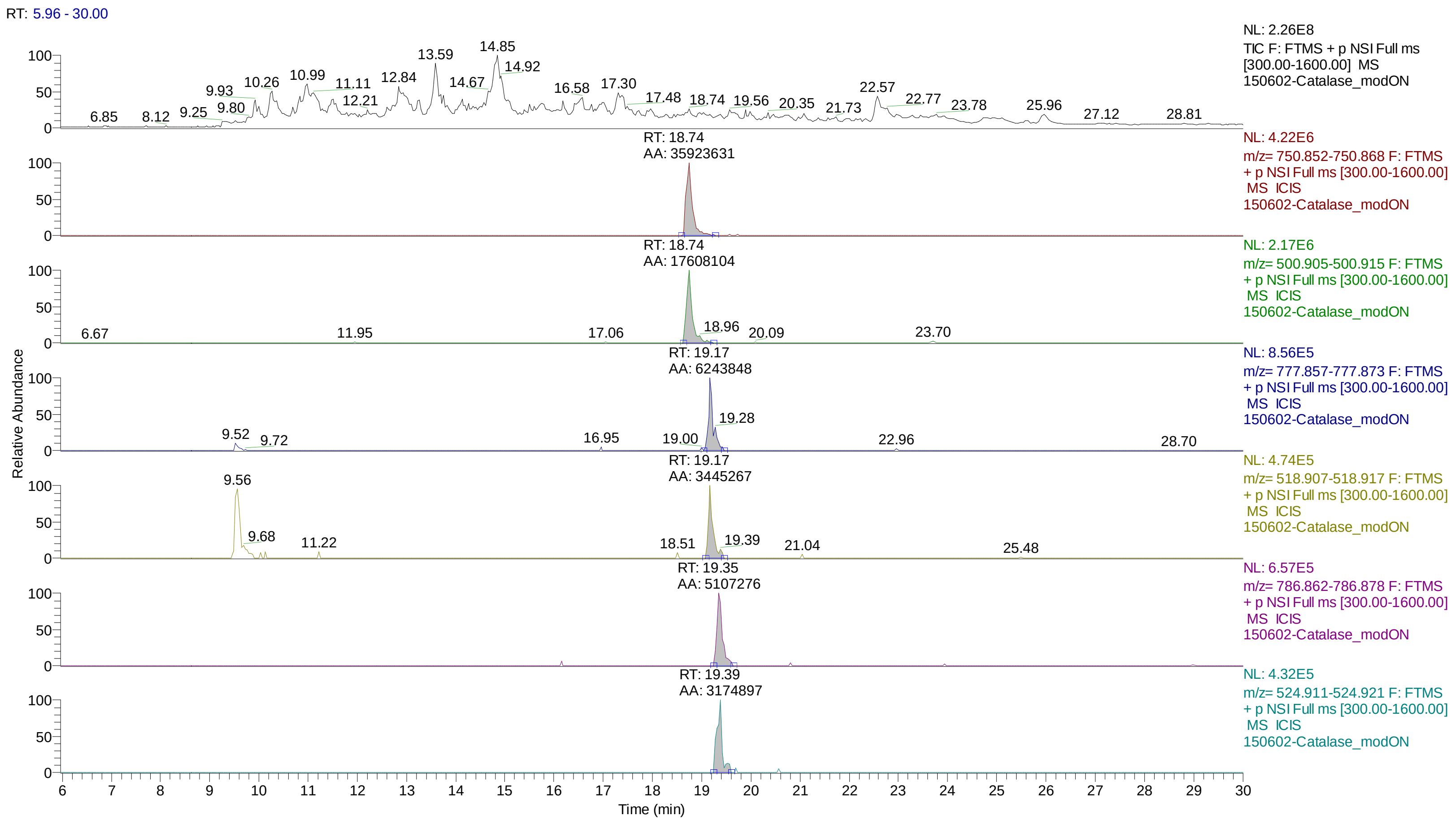

Fig. AA 83-94 in sample catalase (modified), top to bottom:

**83 - 94**	**750.8597**	**F.GYFEVTHDITRY.S**		
**83 - 94**	**500.9095**	**F.GYFEVTHDITRY.S**		
**83 - 94**	**777.8653**	**F.GYFEVTHDITRY.S**	MG-H1 (R)	false positive signal at RT=9.5 min
**83 - 94**	**518.9116**	**F.GYFEVTHDITRY.S**	MG-H1 (R)	false positive signal at RT=9.5 min
**83 - 94**	**786.8702**	**F.GYFEVTHDITRY.S**	Dihydroxyimidazolidine (R)	
**83 - 94**	**524.9158**	**F.GYFEVTHDITRY.S**	Dihydroxyimidazolidine (R)	

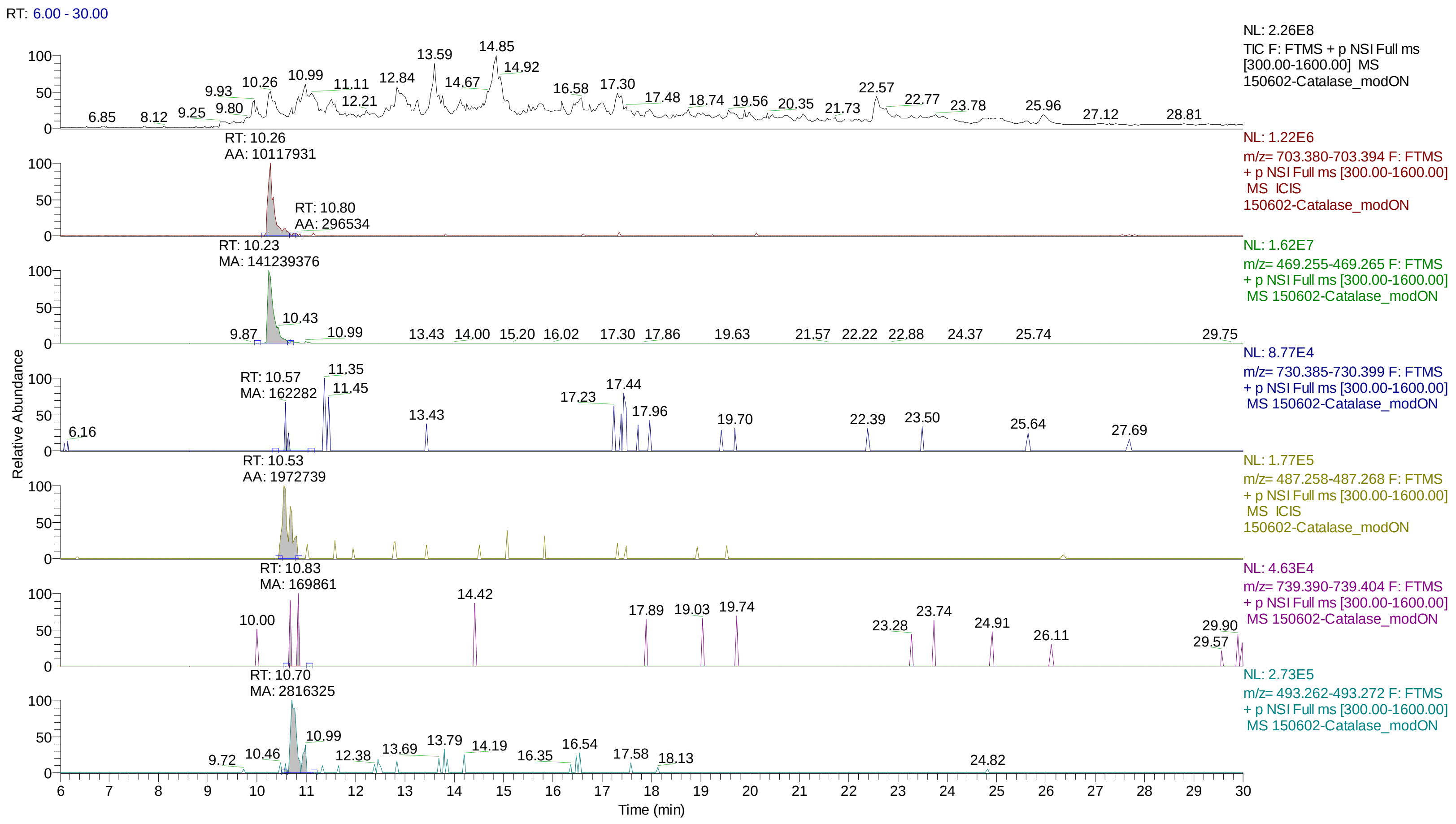

Fig. AA 65-75 in sample catalase (modified), top to bottom:

**65 - 75**	**703.387**	**F.DRERIPERVVH.A**	2+	
**65 - 75**	**469.260**	**F.DRERIPERVVH.A**	3+	
**65 - 75**	**730.392**	**F.DRERIPERVVH.A**	2+	MG-H1 (R)
**65 - 75**	**487.263**	**F.DRERIPERVVH.A**	3+	MG-H1 (R)
**65 - 75**	**739.397**	**F.DRERIPERVVH.A**	2+	Dihydroxyimidazolidine (R)
**65 - 75**	**493.267**	**F.DRERIPERVVH.A**	3+	Dihydroxyimidazolidine (R)

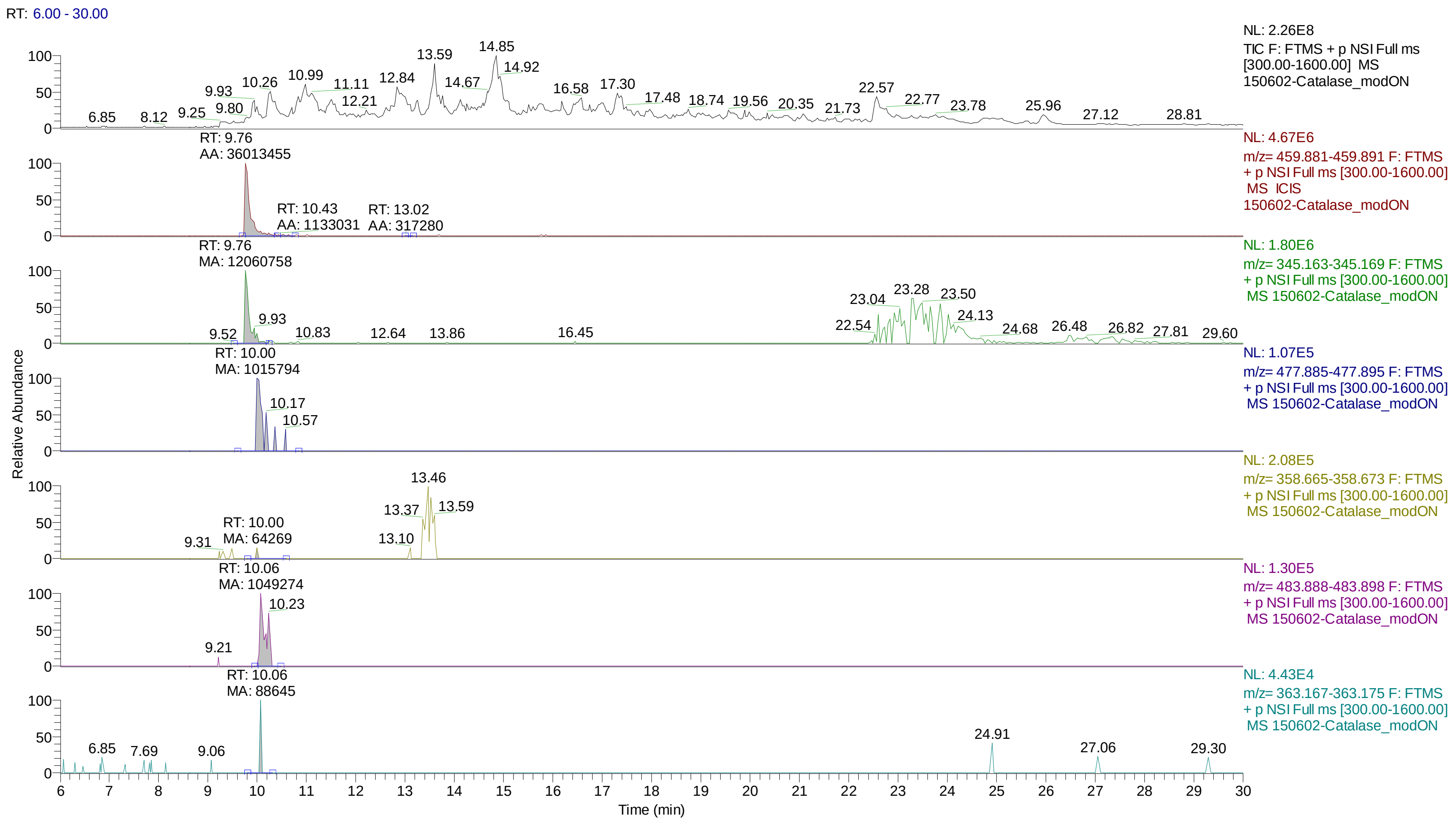

Fig. AA 201-212 in sample catalase (modified), top to bottom:

**201 - 212**	**459.886**	**F.SDRGIPDGHRHM.N**	3+	
**201 - 212**	**345.166**	**F.SDRGIPDGHRHM.N**	4+	
**201 - 212**	**477.889**	**F.SDRGIPDGHRHM.N**	3+	MG-H1 (R)
**201 - 212**	**358.669**	**F.SDRGIPDGHRHM.N**	4+	MG-H1 (R)
**201 - 212**	**483.893**	**F.SDRGIPDGHRHM.N**	3+	Dihydroxyimidazolidine (R)
**201 - 212**	**363.171**	**F.SDRGIPDGHRHM.N**	4+	Dihydroxyimidazolidine (R)

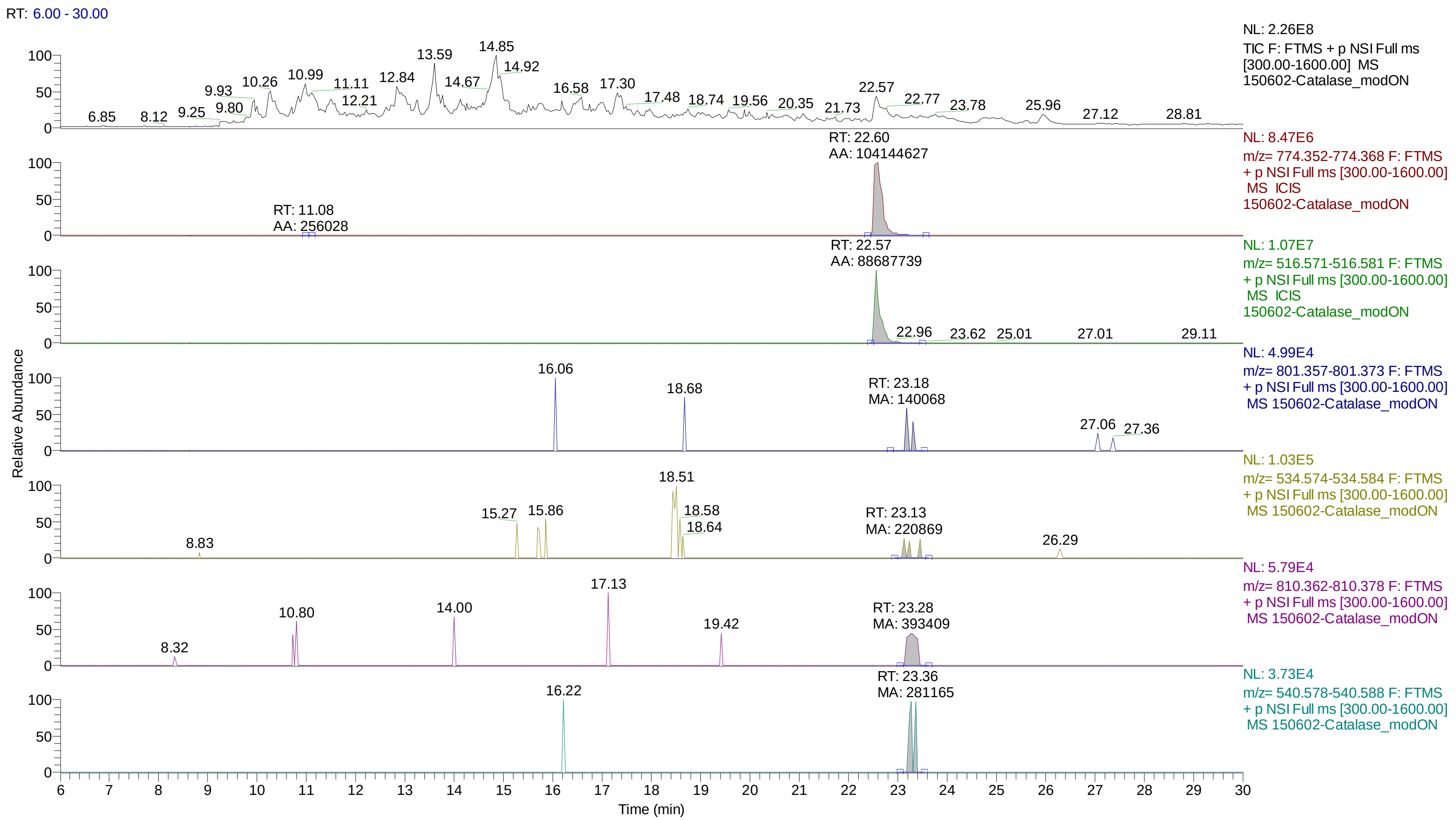

Fig. AA 254-266 in sample catalase (modified), top to bottom:

**254 - 266**	**774.360**	**L.AHEDPDYGLRDLF.N**	2+	
**254 - 266**	**516.576**	**L.AHEDPDYGLRDLF.N**	3+	
**254 - 266**	**801.365**	**L.AHEDPDYGLRDLF.N**	2+	MG-H1 (R)
**254 - 266**	**534.579**	**L.AHEDPDYGLRDLF.N**	3+	MG-H1 (R)
**254 - 266**	**810.370**	**L.AHEDPDYGLRDLF.N**	2+	Dihydroxyimidazolidine (R)
**254 - 266**	**540.583**	**L.AHEDPDYGLRDLF.N**	3+	Dihydroxyimidazolidine (R)

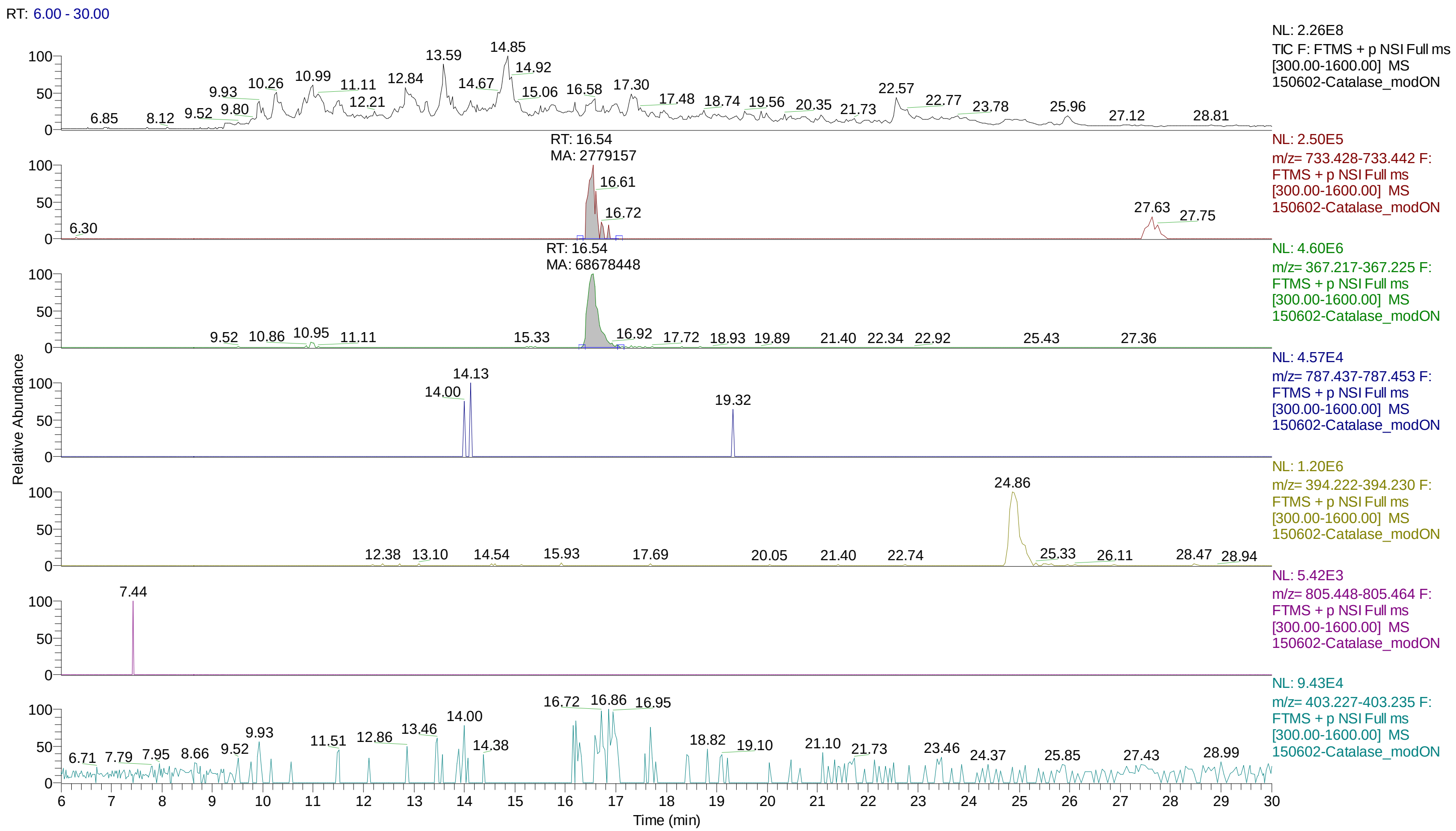

Fig. AA 351-356 in sample catalase (modified), top to bottom:

**351 - 356**	**733.435**	**M.LQGRLF.A**	1+	
**351 - 356**	**367.221**	**M.LQGRLF.A**	2+	
**351 - 356**	**787.445**	**M.LQGRLF.A**	1+	MG-H1 (R)
**351 - 356**	**394.226**	**M.LQGRLF.A**	2+	MG-H1 (R)
**351 - 356**	**805.456**	**M.LQGRLF.A**	1+	Dihydroxyimidazolidine (R)
**351 - 356**	**403.231**	**M.LQGRLF.A**	2+	Dihydroxyimidazolidine (R)

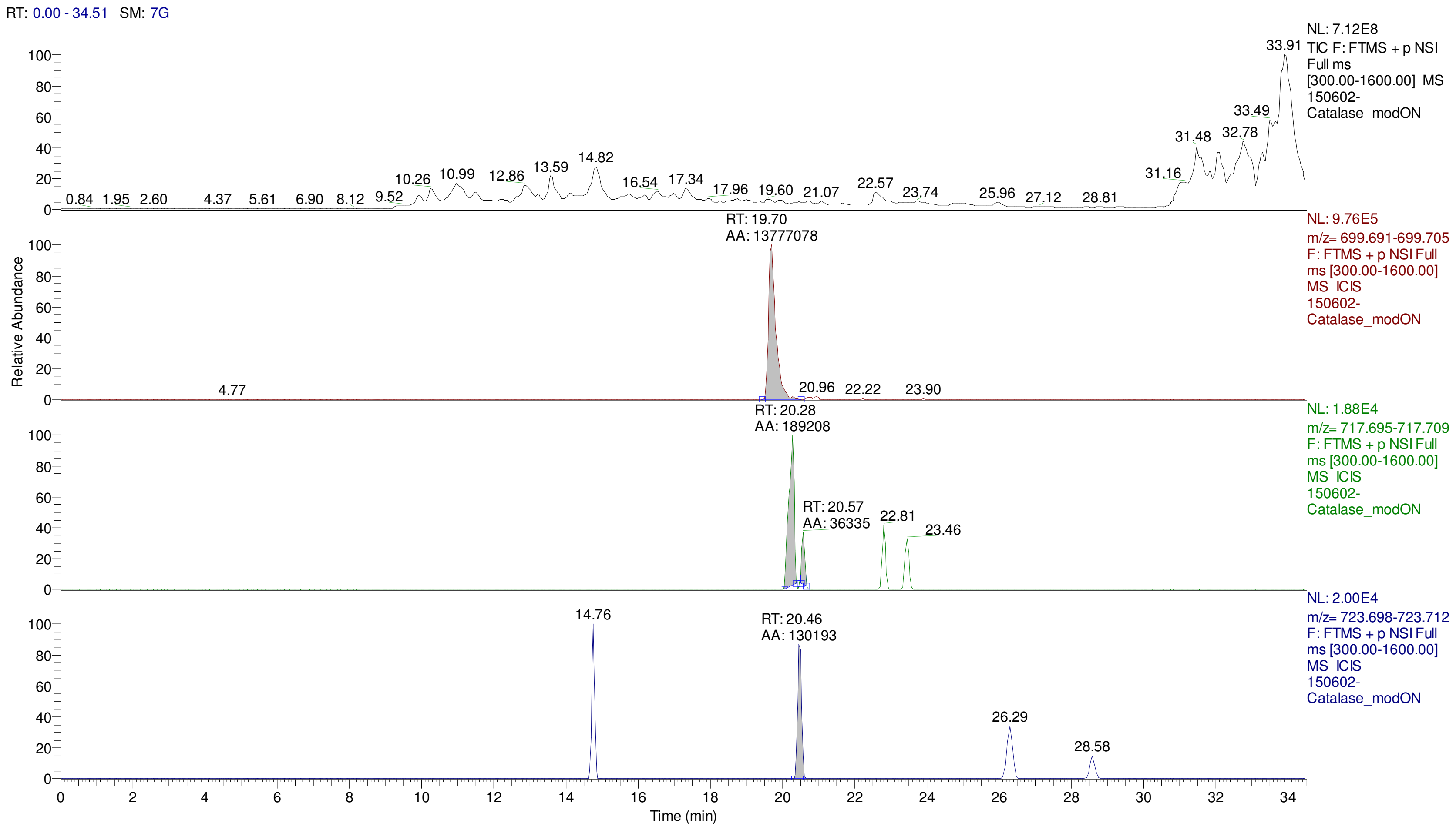

363-379 TIC, unmodified peptide ion, MG-H1 (R), Dihydroxyimidazolidine (R)

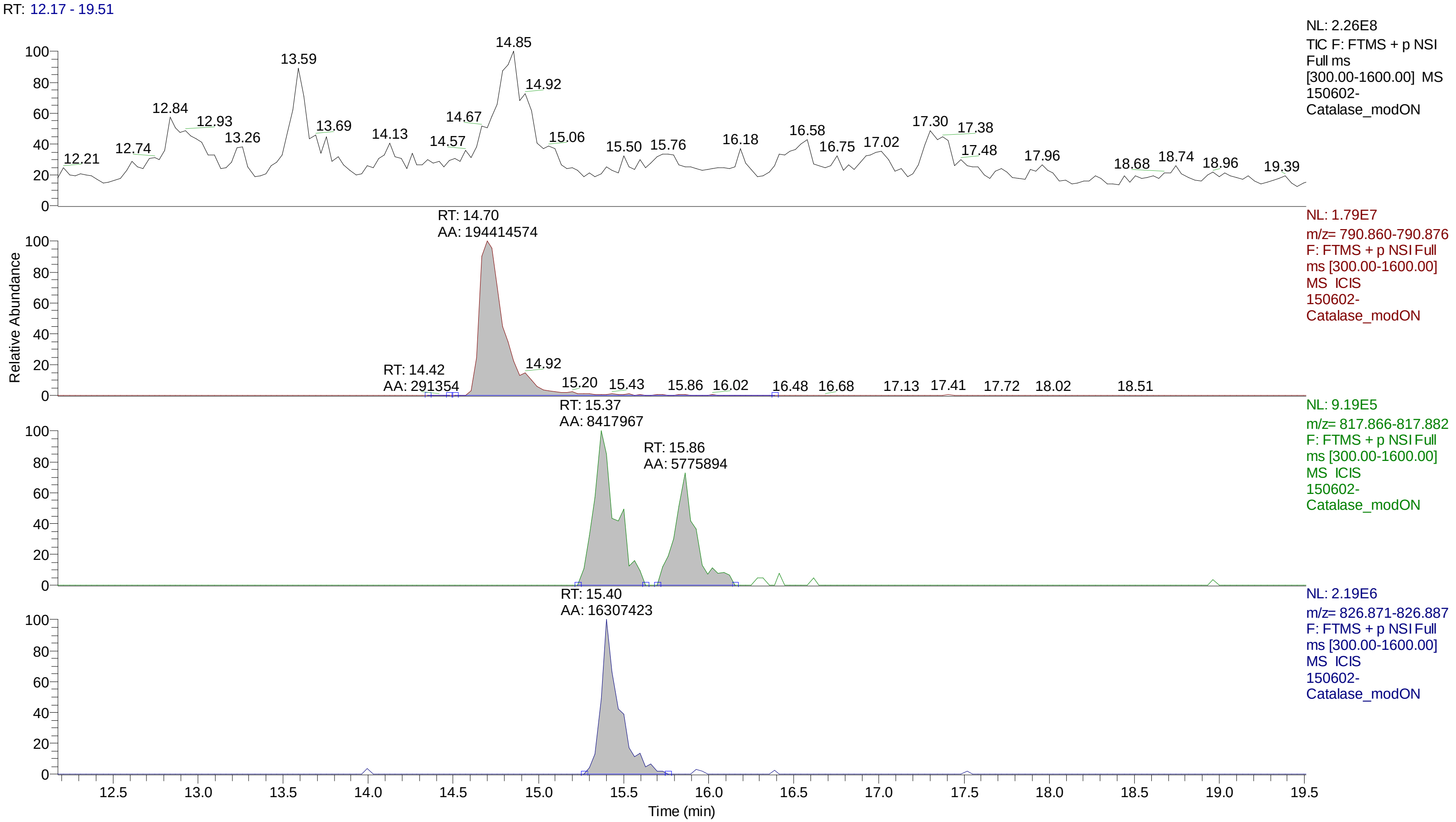

Fig. AA 433-446 in sample catalase (modified), top to bottom:

**433 - 446**	**790.868**	**F.NSANDDNVTQVRTF.Y**	2+	
**433 - 446**	**817.874**	**F.NSANDDNVTQVRTF.Y**	2+	MG-H1 (R)
**433 - 446**	**826.879**	**F.NSANDDNVTQVRTF.Y**	2+	Dihydroxyimidazolidine (R)
